# Improving the quality of mental health services using patient outcome data: making the most of HoNOS

**DOI:** 10.1192/pb.bp.116.054346

**Published:** 2017-06

**Authors:** Mike J. Crawford, Mo Zoha, Alastair J. D. Macdonald, David Kingdon

**Affiliations:** 1 College Centre for Quality Improvement, Royal College of Psychiatrists, London, UK; 2 Central and North West London NHS Foundation Trust, London, UK; 3 South London and Maudsley NHS Foundation Trust, London, UK; 4 Southern Health NHS Foundation Trust, Southampton, UK

## Abstract

Efforts to assess and improve the quality of mental health services are often hampered by a lack of information on patient outcomes. Most mental health services in England have been routinely collecting Health of the Nation Outcome Scales (HoNOS) data for some time. In this article we illustrate how clinical teams have used HoNOS data to identify areas where performance could be improved. HoNOS data have the potential to give clinical teams the information they need to assess the quality of care they deliver, as well as develop and test initiatives aimed at improving the services they provide.

A commitment to improving the quality of healthcare is central to the aims of the National Health Service (NHS).^1^ This commitment involves developing and evaluating new interventions and treatments, obtaining feedback from patients and learning from mistakes.^2^ It also involves monitoring and improving patient outcomes. Despite repeated calls for greater use of patient outcome measures in mental health, available evidence suggests that very few services use them to monitor change over time.^3^ There are a number of important barriers which make it difficult to implement systems for monitoring patient outcomes, including access to reliable and valid measures, the need to protect patient confidentiality and the time and money needed to collect, analyse and report data.^4,5^

While many initiatives aimed at improving the quality of NHS services have been ‘top-down’, it is widely acknowledged that front-line clinicians have a key role in efforts to improve service quality.^6,7^ However, unless clinical teams have access to information about patient outcomes, they cannot assess their performance or identify areas where performance could be improved.

For the past 20 years the Health of the Nation Outcome Scales (HoNOS) have provided a means of assessing the health and social functioning of people who use mental health services.^8^ HoNOS is a clinician-rated outcome measure comprising 12 scales covering symptoms, functioning, social relationships and environmental issues. Each domain is rated by the treating clinician on the scale of 0 to 4: 0 means no problem, 1 means a problem that probably requires no intervention and 2, 3 and 4 correspond to ‘mild’, ‘moderate’ and ‘severe’ problems. They are rated by staff using all available information – not as a questionnaire or interview – based on the worst state in the reference period, usually 2 weeks. There is a glossary, and training in their use is generally recommended.^9^ Although it is possible to calculate a total HoNOS score for a patient, individual scores on each of the 12 scales provide a better guide to the problems they are experiencing and targets for future interventions and treatments. Originally developed to measure the health and social functioning of working-age adults with severe mental illness, the scales have been modified to assess mental health of older adults (HoNOS65+),^10^ children and adolescents (HoNOSCA),^11^ people with intellectual disability (HONOS-LD),^12^ in secure settings (HoNOS-Secure)^13^ and with acute brain injury (HoNOS-ABI).^14^

Use of HoNOS in mental health services in England was patchy until work started on the development of a commissioning tariff based on a Mental Health Clustering Tool, which needed HoNOS scores to be completed on all patients who are in scope of the mental health tariff.^15^ While these plans are still in development, this initiative has led to widespread use of HoNOS throughout the country. In recent years clinicians have begun to consider how these data might be used to assess and improve the quality of care they provide. In the next section we present how clinical teams in three trusts have used HoNOS data to identify problems with the care they provide and plan ways to improve it.

## HoNOS use – examples of application

### Example 1: using HoNOS to examine reasons for admission

Reasons for admission to hospital or to crisis resolution/home treatment (CRHT) teams are poorly understood yet very important in terms of ensuring that available resources are used effectively. As the number of beds decreases, thresholds for admission are becoming increasingly important to assess at a service level.

A team in Southampton used routine HoNOS data to explore mental health problems (such as psychotic symptoms, suicidality and aggression) experienced by adults who were admitted to in-patient units and people referred to CRHT services. They compared the proportion of people who had problems requiring intervention (a score of 2 or more on different HoNOS items) among 3409 people admitted to hospital and 2991 referred to local crisis teams (Table 1). The most prevalent problems among people referred to either service were suicidality and agitation, with levels of agitation higher among those admitted to hospital. Nonetheless, clinicians were surprised to see that only around half of patients admitted to hospital and 39% taken on by crisis teams scored as requiring intervention for suicidality and/or agitation. Even when people with significant problems with psychosis or accommodation status were included, a significant minority did not appear to have major problems requiring intervention.

**Table 1:** HoNOS scale differences between hospital and crisis team admissions

Scores > 2 on HoNOS items	Hospital *n* = 3409 %	Crisis team *n* = 2991 %
1: Agitation	29	16

2: Suicidality	22	27

3: Accommodation	6	5

4: Delusions and hallucinations	13	9

1 or 2	47	39

1 or 2, 3 or 4	66	53

When these findings were discussed within teams, clinicians raised the possibility that people may be being referred to in-patient or CRHT services because of a combination of different problems at less severe level or that staff were under-scoring these items. It also led to discussions about the level of severity at which people were being referred to these services. Discussions based on this information led to a review of in-patient services (numbers of beds in the area were higher than in other comparable catchment areas),^16^ and a review of thresholds for access to CHRT services.

### Example 2: outcomes of patients treated by assessment and brief treatment teams

**Fig. 1:**
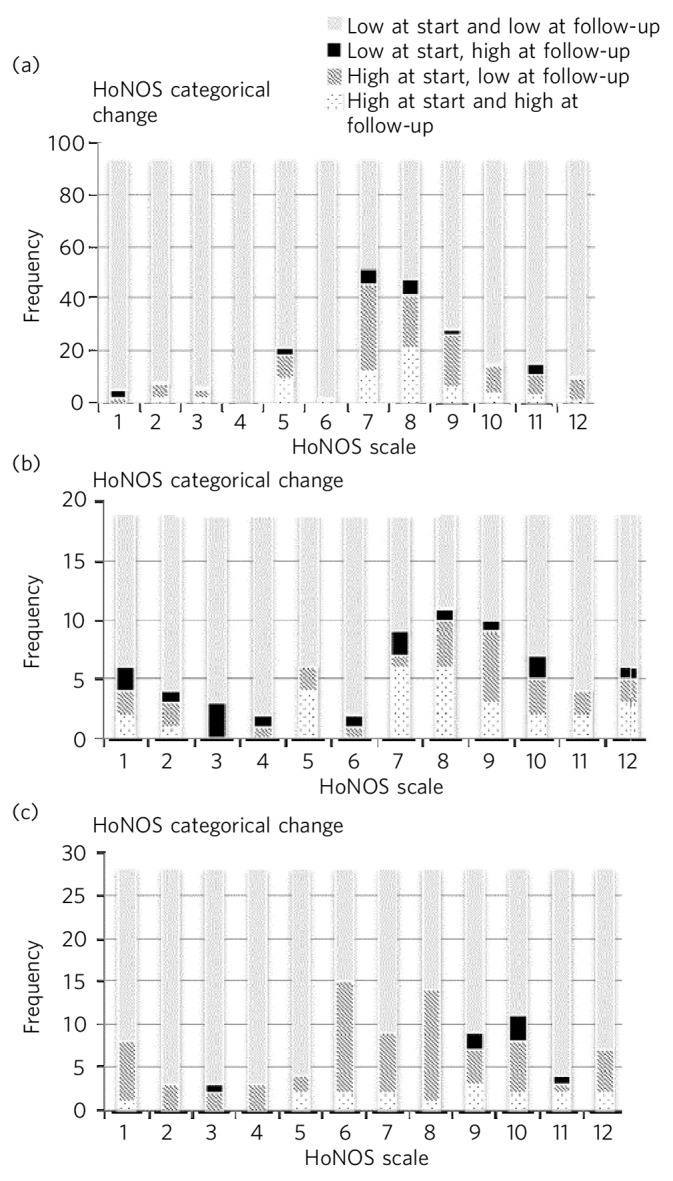
Changes in HoNOS scores among people treated by assessment and brief treatment teams. (a) Clusters 1–5; (b) Clusters 6–8; (c) Clusters 10–15.

Community mental health teams in central London used routine data from HoNOS to examine outcomes of treatment. Changes in mean HoNOS scores were calculated for patients under the care of assessment and brief treatment teams between April 2013 and September 2014 by comparing the mean severity from initial review with that from a follow-up. Scores of 3 (moderate) or 4 (severe) were categorised as ‘high’ and scores of 0 (absent), 1 (minimal) or 2 (mild) were categorised as ‘low’, and proportions of people moving between low and high scores were plotted (Fig. 1). In Fig. 1 differences in severity of each subscale of HoNOS are presented for people in clusters 1–5 (single non-psychotic episode), clusters 6–8 (enduring non-psychotic) and clusters 10–15 (psychosis). The data showed that a smaller proportion of people in clusters 6 to 8 had made progress during their time with teams; among people in clusters 6 to 8, fewer who had high scores at baseline had lower levels at follow-up, especially compared with people in clusters 10 to 15. When data were examined from four other sector services in the trust a similar pattern emerged, with a greater proportion of patients in clusters 6 to 8 failing to show evidence of improvement or problems becoming more severe between the two time points compared with people in clusters 10 to 15.

When these data were presented to front-line staff they commented that it can be difficult to help meet the needs of people in clusters 6–8 (predominately people with personality disorder) through the types of interventions available to staff working in assessment and brief treatment teams. Although staff working in these services are able to refer patients to a local specialist personality disorder service, many do not want the group-based psychological treatment offered by this service or are too chaotic and poorly motivated to engage in psychological treatment. Discussions prompted by a review of these data led to the development and piloting of a six-session brief intervention package for people with personality disorder offered by members of the local specialist team (details available from the authors on request). This package of treatment is based on National Institute for Health and Care Excellence (NICE) guidelines^17^ and focuses on psychoeducation and skills training. It is hoped that some people who initially reject the offer of longer-term psychological treatment can be engaged through this extended assessment and that others will benefit more from this approach than they do from the care they are currently receiving.

### Example 3: comparing outcomes of older adults admitted to in-patient units

**Fig. 2:**
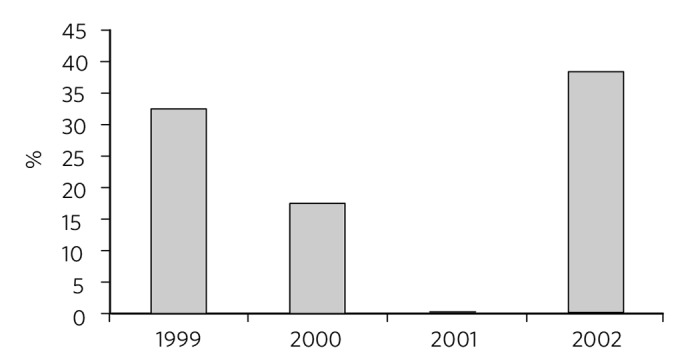
Mean percentage improvement in HoNOS65+ depression scale among patients admitted to an older adult mental health unit.

Staff working on an in-patient mental health unit for older adults with dementia and other organic conditions used routine HoNOS65+ data to examine outcomes of people admitted to their service. It was noted that over a 3-year period the mean percentage improvement in scores on the depression scale of the HoNOS65+ declined (Fig. 2). Outcomes can only be properly understood with reference to context and interventions. These data were therefore compared with those from a similar unit in the same trust with the same operational policy, lengths of stay, diagnostic and demographic characteristics, and initial severity scores. Data from this unit showed that mean percentage improvement on the depression scale over the same period was approximately 50%. The team did not have and still do not have direct data on interventions, but in 2001 there was a pilot study of the systematic recording of care plans, and these data were linked to outcomes data. It transpired that in the unit with the poorer outcomes, all patients with dementia were automatically given night-time benzodiazepine hypnotics. Furthermore, there was a strong association between being given night-time benzodiazepines and poor outcomes. During discussion with the teams it was agreed that routine use of benzodiazepines was a plausible explanation of poor outcomes and this policy was revoked. Over the course of the next year mental health outcomes of patients admitted to the unit improved (Fig. 2).

## Discussion

The examples given above illustrate how front-line clinical teams have used routinely collected HoNOS data to examine and to try to improve the outcomes of the patients they treat. While changes to mental health services will continue to be made in response to new national policy directives, new research findings and new technologies, we believe that one of the most effective ways to improve service quality is ‘bottom-up’: through local teams using local data to drive change. However, front-line clinicians face a number of significant challenges when trying to assess and improve the quality of the care they provide. Chief among these are limited time and other resources needed to collect data on patient outcomes. We are aware of numerous occasions when clinical teams have made changes to the services they provide but have not had the resources to examine whether these changes led to improvements in patient care. In other instances, baseline audits are conducted that identify problems in a service that teams try to correct, but staff have not had time to assess whether these changes benefited patients. To fulfil the NHS promise to patients to continuously work to improve service quality, clinical teams need to be able to access data on patient outcomes. Yet the experience of participants in the UK Routine Clinical Outcomes Network (www.ukrcom.org) suggests that very few services provide outcomes data to their teams. Embarking on new efforts to collect patient- and staff-rated outcomes is expensive and time consuming. By contrast, routinely collected HoNOS data in England provide an important source of clinician-rated patient outcomes that do not require additional resources to be spent and can be used to assess and improve the quality of care that teams provide.

### Challenges to widespread HoNOS use

While the vignettes above illustrate how HoNOS data have been used by front-line clinical teams, a number of obstacles need to be overcome if this approach is to become more widespread.

First, concerns have been raised about the quality of routine HoNOS data.^18^ Available evidence suggests that if staff are provided with appropriate support and training, HoNOS can be used to generate reliable information that can be used to compare different services and examine changes in patient outcomes over time.^19^ Second, IT systems in trusts need to be able to generate reports on outcome data in a form that clinical teams find useful. Third, data from HoNOS and other routine outcomes scales need to be interpreted cautiously. Random variation and subtle changes in practice and case-mix may have led to changes in patients outcomes over time. Separating real and spurious differences can be difficult.^20^ Finally, teams need to be given time and space to examine their data, learn from them and use them to evaluate their efforts to improve service quality. If staff are supported to generate reliable data and systems are available to deliver data to front-line clinical teams, then these data have the potential to be used in clinical audit and in alternative models for improving service quality, such as Plan–Do–Study–Act cycles.^21^ The latter approach may have some advantages over traditional audits because it allows the impact of changes in practice to be examined more frequently and provides a more iterative approach to developing changes aimed at improving patient outcomes.^22,23^

At present, most staff see collecting outcome data as an ‘invisible task’, in which time is spent collecting and entering data for no purpose.^24^ If systems can be implemented that deliver feedback to staff on service-level patient outcomes, staff are more likely to value collecting these data. For instance, in South London and Maudsley NHS Foundation Trust and Central and North West London NHS Foundation Trust, clinicians have organised meetings for staff in which HoNOS data are presented and discussed. Feedback from staff attending these meetings has shown they value getting this information and their comments have been used to refine the way that data are collated and presented (most staff indicated a preference for the categorical change model presented in Fig. 1 rather than changes in total HoNOS scores).

While HoNOS scores collected through the current mental health payment initiative^15^ provide a rich source of routine data on patient outcomes, the timing of assessments is unlikely to be optimal for evaluating the impact of treatments and services. Further work is needed to establish when outcome assessments are best undertaken in different settings to compare services and assess the impact of quality improvement initiatives.

HoNOS data are not the only form of evidence that mental health services collect. For instance, psychiatric in-patients are asked to complete the ‘friends and family test’ (a two-item short patient-rated experience measure).^25^ However, there is very little evidence that these data are being fed back to clinicians to allow them to reflect on differences in levels of patient satisfaction over time or between different teams.^26^ Such data also have the potential to stimulate bottom-up efforts to assess and improve service quality if steps are taken to use them in this way. One of the great strengths of HoNOS data is that they provide a summary of mental health, behavioural problems and social factors. Although this means that HoNOS can be used under circumstances where poor mental health or impaired cognition may limit the value of patient-rated data, there are drawbacks to relying solely on clinician-rated outcomes. The possibility that outcome data could be used to pay services based on patient outcomes could paradoxically reduce their value as a means to assess and improve service quality.^27^ This is commonly known as Goodhart's law after the British economist Charles Goodhart: ‘When a measure becomes a target, it ceases to be a good measure’.^28^

Mental health trusts in England are currently collecting large amounts of outcome data using HoNOS. We believe that efforts by mental health services to use HoNOS data and other routinely collected patient outcomes have the potential to make better use of available resources and engage front-line clinicians in efforts to improve patient outcomes.
